# MicroRNA-200b regulates distal airway development by maintaining epithelial integrity

**DOI:** 10.1038/s41598-017-05412-y

**Published:** 2017-07-25

**Authors:** Naghmeh Khoshgoo, Robin Visser, Landon Falk, Chelsea A. Day, Dustin Ameis, Barbara M. Iwasiow, Fuqin Zhu, Arzu Öztürk, Sujata Basu, Molly Pind, Agnes Fresnosa, Mike Jackson, Vinaya Kumar Siragam, Gerald Stelmack, Geoffrey G. Hicks, Andrew J. Halayko, Richard Keijzer

**Affiliations:** 1 Biology of Breathing Group, The Children’s Hospital Research Institute of Manitoba, Winnipeg, Manitoba Canada; 2 Departments of Surgery, Division of Pediatric Surgery and Pediatrics & Child Health, University of Manitoba, Winnipeg, Manitoba Canada; 3 Department of Physiology and Pathophysiology, University of Manitoba, Winnipeg, Manitoba Canada; 4 Research Institute of Oncology and Hematology, CancerCare Manitoba, Winnipeg, Manitoba Canada; 5 Department of Biochemistry & Medical Genetics, University of Manitoba, Winnipeg, Manitoba Canada; 6 Small Animal and Materials Imaging Core Facility, University of Manitoba, Winnipeg, Manitoba Canada

## Abstract

miR-200b plays a role in epithelial-to-mesenchymal transition (EMT) in cancer. We recently reported abnormal expression of miR-200b in the context of human pulmonary hypoplasia in congenital diaphragmatic hernia (CDH). Smaller lung size, a lower number of airway generations, and a thicker mesenchyme characterize pulmonary hypoplasia in CDH. The aim of this study was to define the role of miR-200b during lung development. Here we show that miR-200b^−/−^ mice have abnormal lung function due to dysfunctional surfactant, increased fibroblast-like cells and thicker mesenchyme in between the alveolar walls. We profiled the lung transcriptome in miR-200b^−/−^ mice, and, using Gene Ontology analysis, we determined that the most affected biological processes include cell cycle, apoptosis and protein transport. Our results demonstrate that miR-200b regulates distal airway development through maintaining an epithelial cell phenotype. The lung abnormalities observed in miR-200b^−/−^ mice recapitulate lung hypoplasia in CDH.

## Introduction

Every year, over 50,000 children are born with congenital diaphragmatic hernia (CDH) associated with abnormal lung development resulting in lung hypoplasia and persistent pulmonary hypertension^1–3^. CDH occurs as frequently as cystic fibrosis, but the pathogenesis is poorly understood. Smaller lung size, lower number of airway generations and a thicker mesenchyme characterize the abnormal lungs in CDH^1^. We have previously shown an inherent lung development defect in CDH^4^.

Lung development is a continuous process comprised of five developmental stages: embryonic, pseudo-glandular, canalicular, terminal saccular and alveolar^5^. Functionally, the early two stages are characterized by lung branching morphogenesis. Cell specification, vascularization and reduction of mesenchyme to form thin air-blood interfaces for gas exchange characterize the later stages^6, 7^.

MicroRNAs (miRNA) are small, non-coding RNAs that regulate gene expression through mRNA stability and translation^8–10^. They are essential for development and homeostasis of organs^11–14^. More than 1800 microRNAs have been identified in human^15^. Research focusing on the role of microRNAs in lung development and disease is limited. We recently discovered that miR-200b is elevated in abnormal lungs of human CDH babies. In the same study, we found that higher miR-200b expression in the fetal tracheal fluid of CDH fetus is associated with a better response to fetoscopic endoluminal tracheal occlusion (FETO, a prenatal therapy to promote lung growth)^16^.

MiR-200b belongs to the miR-200 family (miR-141, miR-429, miR-200a, miR-200b and miR-200c) and regulates epithelial-to-mesenchymal transition (EMT) in cancer and organ fibrosis^17–20^. Others have shown that miR-200 is down-regulated in a mouse model of fibrotic lung disease and human patients with idiopathic pulmonary fibrosis (IPF)^21^. Also, miR-200b can inhibit migration and invasion of non-small cell lung cancer cells^22^. The role of miR-200b during normal lung development has yet to be defined.

The goal of this study was to delineate the role of miR-200b during lung development using loss of function models *in vivo*. We generated a miR-200b^−/−^(KO) mouse to evaluate the functional impact of miR-200b absence on development *in vivo*. miR-200b deficient mice had stiffer lungs due to disturbed distal airway branching, thicker alveolar walls and downregulation of epithelial cell differentiation. Our data suggest that miR-200b is required to achieve the necessary balance in development of lung epithelial cells and fibroblasts to ensure development of a structurally and functionally effective respiratory organ.

## Results

### miR-200b is highly expressed during different stages of lung development

**Figure 1:**
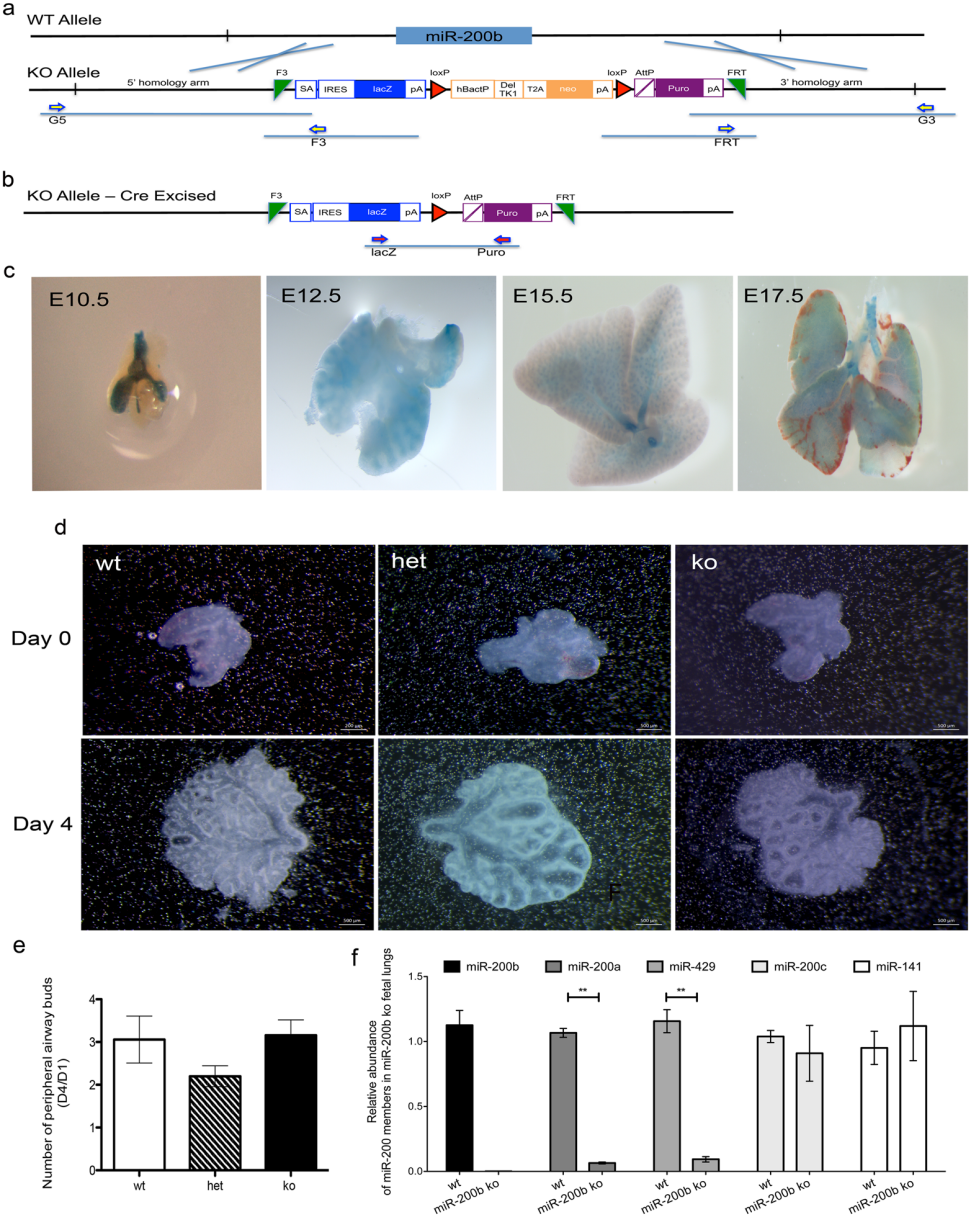
Generation and validation of miR-200b^−/−^ mice (miR200b^tm1.1(NCOM)MFGC^). (**a**) Targeted miR-200b knockout allele. The complete miR-200b non-coding gene (WT allele) was replaced by a NorCOMM cassette via targeted homologous recombination in C57Bl/6N mouse C2 ES cells. The NorCOMM targeting cassette consists of three functional components: a lacZ expression reporter (blue boxes); a loxP flanked (red triangles) hβact promoter driven ∆TK1-T2A-neomycin selectable marker (orange boxes), which can be subsequently excised by cre-recombinase; and a docking cassette AttP-Puro-pA that can be utilized to exchange the entire NorCOMM cassette to any other allele (purple boxes) and once placed, the remaining flanked sequences can be removed by flpO-recombinase between F3 and FRT sites (green triangles) *in vivo*. The length of 5′ and 3′ homology arms are 2891 bp and 6595 bp in the targeting vector, respectively. The targeted miR-200b gene was highlighted in the resulting knockout allele (KO Allele). (**b**) Targeted miR-200b knockout mice. Removal of the hβact promoter driven ∆TK1-T2A-neomycin cassette was performed by mating male miR200b^tm1(NCOM)MFGC^ mice harboring the miR-200b KO allele with female CMV-Cre transgenic mice. Precise cre-excision of the neomycin cassette was determined by PCR and sequence validation, as shown with primers specific to LacZ and Puro cassette by purple arrows in Panel C. The resultant miR-200b cre-excised allele is shown as miR200b^tm1(NCOM)MFGC^. (**c**) LacZ staining of miR-200b^−/−^ mice demonstrated the expression in both epithelial and mesenchymal cells during lung development. (**d,e**) Lung explants culture of E11.5 lung explants did not show significant differences in proximal branching morphogenesis between miR-200b^+/+^, miR-200b^+/−^ and miR-200b^−/−^ lungs. (**f**) RT-qPCR for all miR-200 family members on fetal lung explants using LNA primers. miR-200b absence was confirmed. miR-200a and miR-429 were significantly downregulated, but no changes were observed in abundance of miR-200c and miR-141 **P < 0.01, Student’s t-test, Data represent mean ± SEM of at least four independent experiments.

We generated miR-200b^−/−^ (KO) mice by replacing the complete miR-200b gene with a LacZ-reporter by targeted homologous recombination in C57Bl/6N mouse embryonic stem cells using the NorCOMM cassette (Fig. 1a,b, Supplementary Fig. 1). MiR-200b^+/−^ mice were inter-crossed with the C57Bl/6N mice for at least 8 generations before use in experimental cohort studies. We confirmed complete knockout of miR-200b expression by RT-qPCR in fetal lungs (Fig. 1f) and lungs from 8-week old mice (Supplementary Fig. 5). We showed that mature microRNAs transcribed in the same cluster - miR-200a and miR-429 - were still expressed, albeit lower compared to miR-200b^+/+^ lungs (Fig. 1f, Supplementary Fig. 5). The expression of miR-200c and miR-141 did not change in the miR-200b^−/−^ lungs compared to miR-200b^+/+^ lungs, suggesting that there were no compensatory effects on other family members (Fig. 1f and Supplementary Fig. 5).

Genotyping data from breeding miR-200b^+/−^ x miR-200b^+/−^ mice revealed an expected Mendelian distribution, indicating that these mice experience no embryonic lethality, and they were viable, fertile and appeared morphologically normal. In contrast to miR-200b/miR-429^−/−^ mice reported by others^23^, our miR-200b^−/−^ mice carried a lacZ-reporter gene that we exploited to localize miR-200b promoter activity during development (Fig. 1c). We observed a high expression in the endoderm of lung buds and developing airways during lung development. At E12.5, during branching morphogenesis, we observed lacZ staining mainly in the endodermal cells of the lungs and a faint expression in the mesenchymal cells. Notably, we observed high LacZ-miR-200b expression in other organs where development also hinges on epithelial-mesenchymal interactions and branching morphogenesis. These include the developing inner ear^24^, palate^25^ and mammary buds^26^ (Supplementary Fig. 3), consistent with prior reports suggesting that miR-200b expression is highly coordinated and associated with control of the development of these organs^27–29^.

### miR-200b absence does not affect proximal airway branching

Based on this high and dynamic expression of miR-200b, we focused first on the role of miR-200b during the early stages of lung development. We cultured mutant lungs from E11.5 mice as described before^4^. Branching patterns between the different lung explants were not affected in a significant manner (Fig. 1d,e). To evaluate if the absence of miR-200b resulted in compensatory upregulation of other family members, we assessed the abundance of all family members in fetal lung explants using RT-qPCR (Fig. 1f). Mature microRNAs transcribed in the same cluster–miR-200a and miR-429–were still expressed, albeit lower compared to miR-200b^+/+^ lungs (wt). The expression of miR-200c and miR-141 did not change in the miR-200b^−/−^ fetal lungs compared to miR-200b^+/+^ lungs, suggesting that there were no compensatory effects on other family members. Newborn miR-200b^+/−^ and miR-200b^−/−^ mice did not display any breathing difficulties after birth, and together, these results indicate that proximal airway branching is not influenced by absence of miR-200b during development.

### miR-200b^−/−^ mice have higher lung tissue damping and elastance with lower hysteresivity

**Figure 2:**
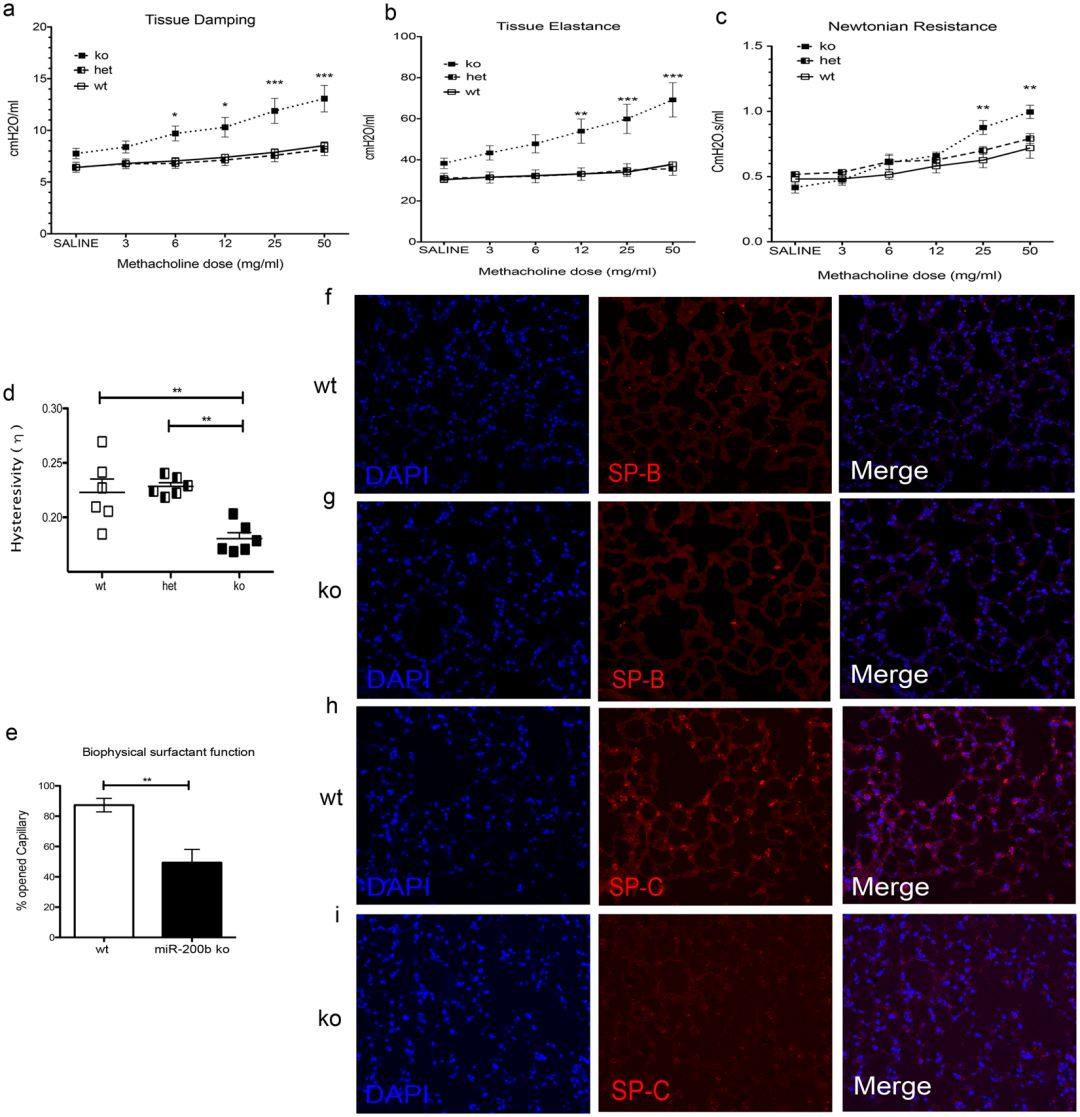
miR-200b is required for normal lung function. (**a**) Lung mechanics studies performed on 8-week-old mice demonstrated that miR-200b^−/−^ (ko) mice have higher lung tissue resistance (damping) when challenged with 6 mg/ml methacholine or higher (**b**) miR-200b ko mice have higher lung tissue stiffness (Elastance) at 12, 25 and 50 mg/ml of methacholine (**c**) and more conducting airway resistance at high concentrations of methacholine. (**d**) Accessing lung hysteresivity at the time of saline challenge (before methacholine challenge) showed lower hysteresivity in miR-200b ko mice. *P < 0.05, **P < 0.01, ***P < 0.001, two-way ANOVA. Data represent mean ± SEM of at least six independent experiments. (**e**) Biophysical surfactant function of miR-200b ko lungs (8-week-old) measured using capillary surfactometry. MiR-200b^−/−^ mice have decreased surfactant function compared to wt. **P < 0.01, Student’s t-test, Data represent mean ± SEM of at least three independent experiments. (**f**,**g**) Immunofluorescence of 8-week-old wt (**f**) and ko (**g**) lungs showed decreased abundance of Surfactant Protein-B (SP-B) in ko lungs. (**h**,**i**) Immunofluorescence of 8-week-old wt (**h**) and ko (**i**) lungs showed decreased abundance of pro-Surfactant Protein-C (SP-C) in ko lungs.

To determine whether lung function is affected in adult miR-200b^−/−^ mice, we performed *in vivo* lung mechanics analyses using a *flexi*VENT small animal ventilator in 8-week-old miR-200b^−/−^ mice and compared these with studies from miR-200b^+/−^ and miR-200b^+/+^ mice. Mice were subjected to increasing doses of nebulized methacholine (MCh) to assess concentration dependent response characteristics of respiratory mechanics. Inhaled methacholine causes constriction of airway smooth muscle cells (bronchoconstriction)^30^. At rest, miR-200b^−/−^ mice did not exhibit altered peripheral tissue/airway resistance (tissue damping), however after challenge with methacholine (MCh; 6 mg/ml and higher) a substantive increase in tissue damping was revealed (Fig. 2a). Consistent with development of a low compliance or “fibrotic” lung, miR-200b^−/−^ mice also showed significantly higher tissue elastance upon MCh-challenge (12 mg/ml and higher) (Fig. 2b). At baseline, airflow resistance in conducting airways (Newtonian resistance) was not different between different groups of mice, but was significantly higher in miR-200b^−/−^ mice challenged with high concentrations of MCh (Fig. 2c). Finally, miR-200b^−/−^ mice demonstrated significantly lower hysteresivity (elastic hysteresis) in pressure-volume loops obtained before and after MCh challenge, consistent with the increased elastance we observed in these animals (Fig. 2d, Supplementary Fig. 4). To examine if the absence of miR-200b influenced the expression of other family members and therefore contributed to the observed phenotype, we measured the abundance of all miR-200b family members in 8-week-old wt and miR-200b^−/−^ lungs. Like what we observed in fetal lungs, adult miR-200b^−/−^ lungs had lower miR-200ba and miR-429 abundance, but no changes in miR-200c and miR-141 abundance (Supplementary Fig. 5). We also observed lower miR-200a and miR-429 in kidney tissues of miR-200b^−/−^ mice (Supplementary Fig. 6).

### miR-200b^−/−^ lungs have dysfunctional surfactant and denser parenchyma with more fibroblast- like cells

Some of the lung function abnormalities in miR-200^−/−^ could result directly from changes in surfactant function or distal airway branching. Therefore, we assessed the biophysical function of surfactant from miR-200b^−/−^ and miR-200^+/+^ lungs using capillary surfactometry. We found that the surface tension-reducing capacity of surfactant in miR-200b^−/−^ mice was markedly reduced (Fig. 2e), a finding that correlated with compromised labeling of Surfactant protein-B (SP-B) (Fig. 2f,g) and pro-Surfactant Protein-C (SP-C) in these lung tissues. Our data suggest a functional role for miR-200b in development of surfactant properties, resulting in reduced lung compliance in miR-200b^−/−^ mice.

**Figure 3:**
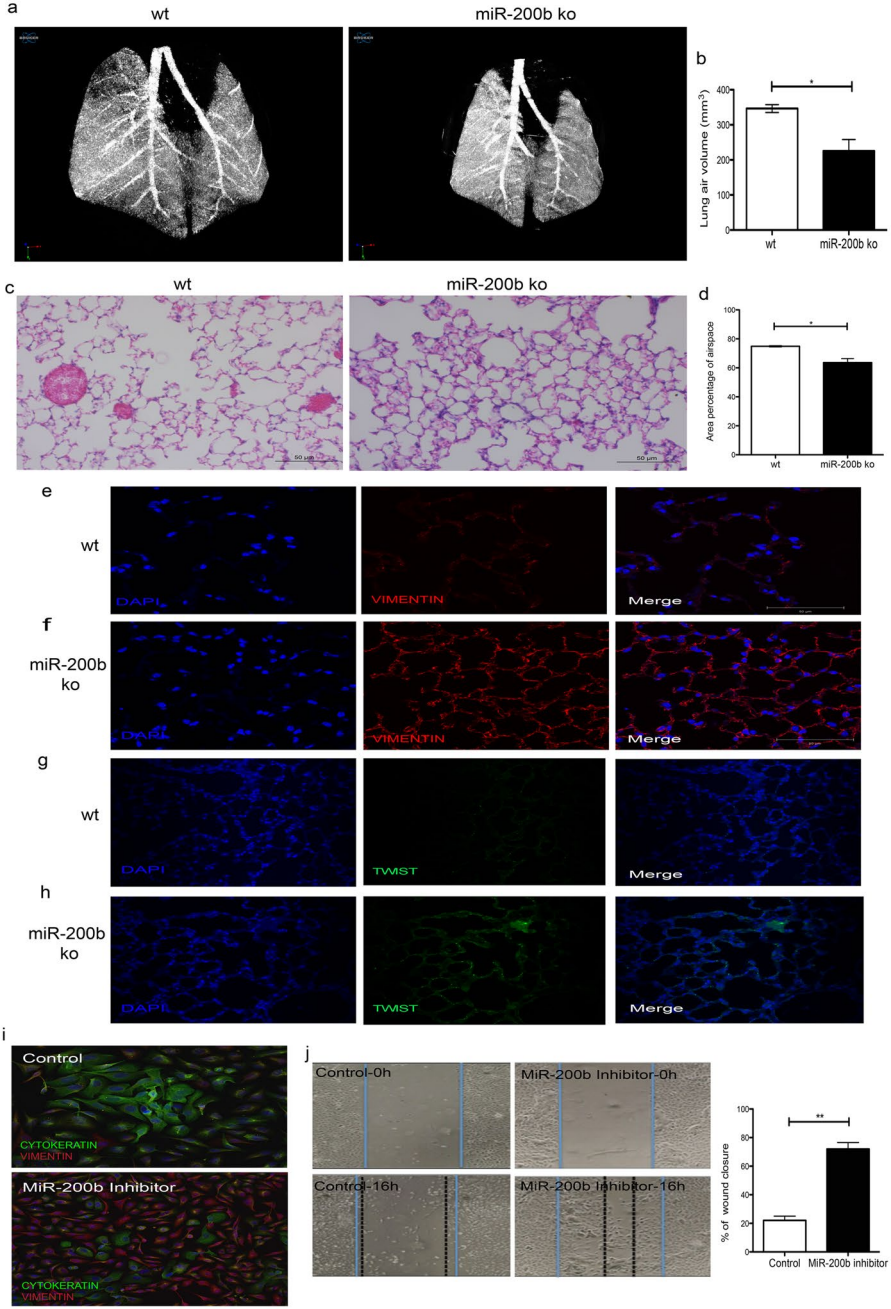
miR-200b knockout mice have denser parenchyma, thickened alveolar walls, lower distal branching and more fibroblast-like cells. (**a**) *In vivo* micro-CT scans of 8-weeks-old mice using the SkyScan 1176 x-ray microtomography system equipped with a large format 11 megapixel x-ray camera. MiR-200b knockout (ko) mice have denser lung parenchyma (gray area in the peripheral area) and a lower number of distal airways (smaller distance between the large airways). (**b**) miR-200b^−/−^ lungs have significantly lower levels of lung air volume than miR-200b^+/+^. Lung airspace volume was measured on alive mice using micro-CT scan. *P < 0.05, Student’s t-test, Data represent mean ± SEM of at least three independent experiments. (**c**) Hematoxylin and eosin (H&E) staining of miR-200b^−/−^ lungs confirmed that these lungs have less septation and thicker alveolar walls when compared to wild type lungs. (**d**) Area percentage of airspace of miR-200b^−/−^ lungs was significantly decreased compare to miR-200b^+/+^ lungs. H&E stained peripheral lung sections were scanned and the percentage of airspace over total lung was measured using ZEN Image Analysis. (**e**,**f**) Immunostaining for Vimentin on wt (**e**) and ko (**f**) lungs demonstrated higher expression of Vimentin protein in ko parenchyma than the wt. (**g**,**h**) Immunofluorescence for Twist protein on wt (**g**) and ko (**h**) demonstrated higher expression of Twist in the ko than the wt. (**i**) Immunostaining of the miR-200b transfected cells after 48 h demonstrated higher expression of Vimentin and lower expression of Cytokeratin in these cells compared to cells transfected with a negative control. (**j**) A wound healing (scratch) assay was performed on BEAS-2B or control cells transfected with miR-200b inhibitors (for 18 h). Pictures were taken at time 0 h when the scratch was made and 18 h later and the migration rates were calculated (the difference between distance from the right to left border at 16 h divided by the distance from the right to left border at the start time).

We evaluated lung morphometry in miR-200b^−/−^ mice in a quantitative fashion, using two different, complementary approaches. We first performed *in vivo* high-resolution micro-CT scanning on live animals to eliminate the effects of tissue processing and inflation on the morphometry of the lungs. We made reconstructions of the lungs so that data between mice could be directly compared (i.e. if two images show the same level of grey scale, then they exhibit the same degree of x-ray attenuation). We then set a threshold to segment the data into solid tissue and air (using the same threshold value for all images) and calculated the volume of air in the lungs. To ensure that we used comparable areas of the lungs, we identified the carina in each lung and then moved 3.25 mm above this. We then calculated the air volume from this point to the base of the lungs. Using *in vivo* high-resolution micro-CT scanning, we found that the density of lung parenchyma in miR-200b^−/−^ mice is greater (gray area), and by measuring the airspace volume we demonstrated that distal alveoli are less air-filled compared to lungs from wildtype mice (Fig. 3a,b). This can result from increased alveolar space collapse or smaller alveolar air volume.

Hematoxylin and eosin (H&E) staining of miR-200b^−/−^ lungs that were inflated and embedded in paraffin confirmed that these lungs have fewer septae and thicker alveolar walls when compared to miR-200^+/+^ lungs (Fig. 3c). We then scanned three complete sections per lung of three miR-200b^+/+^ and three miR-200^−/−^ lungs using an Axio Scan.Z1 and used the ZEN Image Analysis software module to calculate the airspace area percentage of each lung. We observed lower percentages of airspace in miR-200^−/−^ lungs (Fig. 3d).

Immunofluorescence studies for vimentin showed that miR-200b^−/−^ lungs have more vimentin-positive cells, suggesting an increased presence of fibroblasts-like, mesenchymal cells (Fig. 3c,d). Also, miR-200b^−/−^lungs showed higher expression of Twist 1 protein – a transcription factor and marker for EMT - compared to miR-200b^+/+^ lungs (Fig. 3e,f). There were no differences in immunofluorescence patterns for other markers of lung fibroblast cell differentiation (Fibroblast growth factor-10) and epithelial cell differentiation (E-cadherin, cytokeratin, and CC-10) (data not shown). Taken together, these results indicate that even though miR-200b^−/−^ mice do not experience obvious breathing difficulties, their lungs display a functional phenotype similar to lung fibrosis and lung hypoplasia observed in children with CDH.

### miR-200b maintained human bronchial epithelial phenotype and function

To evaluate the effect of miR-200b on maintaining human bronchial epithelial cell phenotype, we transfected BEAS-2B cells with miR-200b inhibitors and performed double-immunofluorescence with cytokeratin and vimentin to mark epithelial cells and fibroblasts, respectively. We found that miR-200b inhibitors promoted accumulation of fibroblast cell markers following down regulation of miR-200b (Fig. 3g). To study if this also resulted in functional mesenchymal properties, we performed a scratch wound-healing assay (Fig. 3h). We transfected BEAS-2B cells with miR-200b inhibitors and 18 h later, we observed 25% less scratch wound closure in the control group, whereas, in cultures transfected with miR-200b inhibitors, wound closure was enhanced by 75%.

### mRNA-Seq whole transcriptome analysis demonstrated that mRNAs of epithelial cell differentiation and surfactant genes are most affected in miR-200b^−/−^ lungs

**Figure 4:**
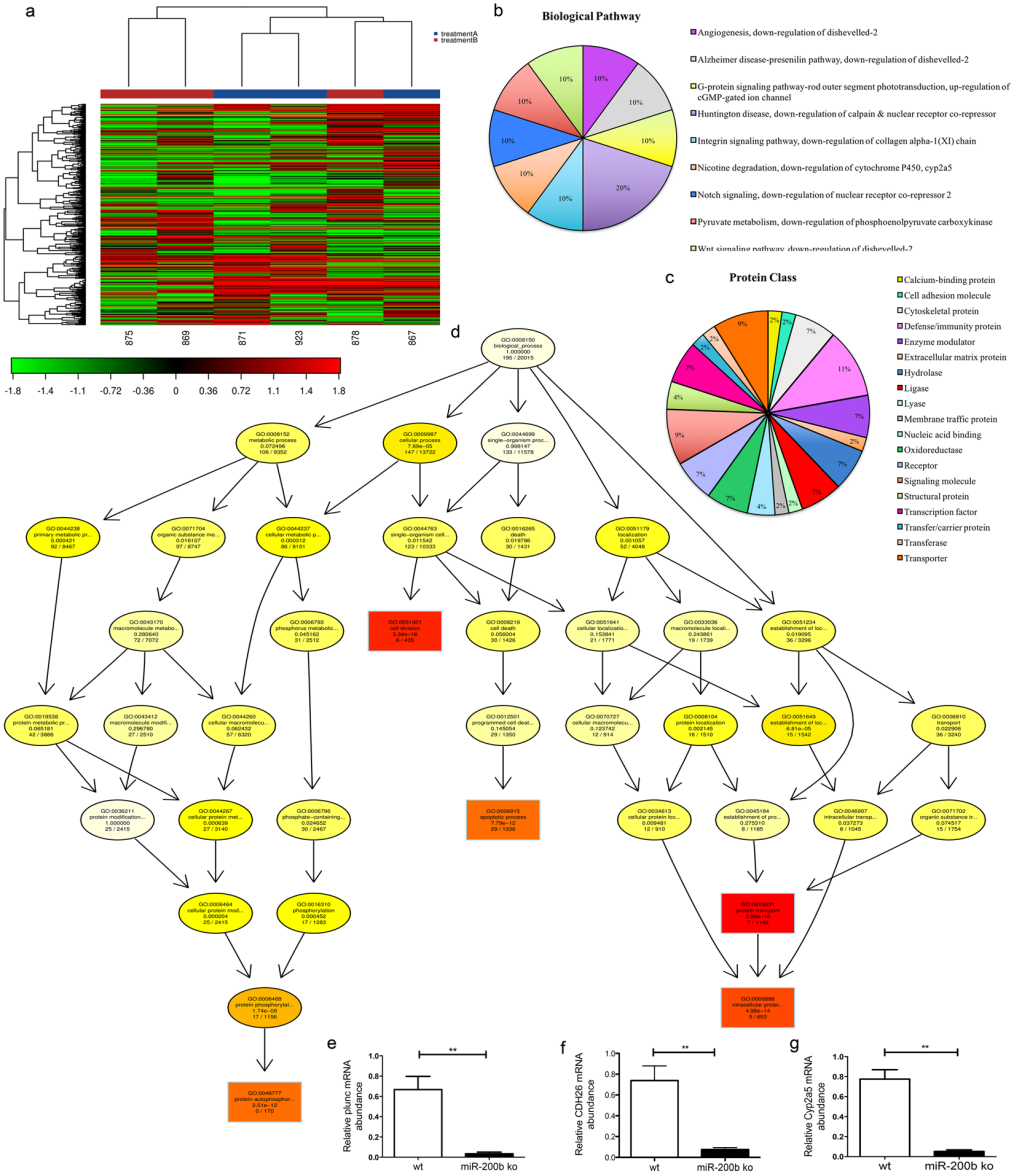
Next Generation Sequencing and Gene ontology (GO) showed the most affected pathways in the lungs of miR-200b^−/−^ mice. (**a**) The heat map diagram shows the results of a two-way hierarchical clustering of RNA transcripts and samples. It includes the 500 genes that have the largest coefficient of variation based on FPKM counts. Each row represents one gene and each column represents one sample. The color represents the relative expression level of a transcript across all samples. The color scale is shown below: red represents an expression level above the mean; green represents an expression level below the mean. (wt samples: 867, 871 and 923; miR-200b ko samples: 875, 878 and 869). (**b**,**c**) Pie charts for transcript gene functional analysis conducted for Biological Pathway and Protein class using the PANTHER gene ontology database. (**d**) GO network generated from the GO terms predicted to be enriched for the Biological process (BP vocabulary). Nodes are colored from red to yellow with the node with the strongest support colored red and nodes with no significant enrichment colored yellow. The five nodes with strongest support are marked with rectangular nodes. (**e**,**f**,**g**) Q-PCR confirmed significantly lower *Plunc*, *Cdh26 and Cyp2a5* mRNA levels in miR-200^−/−^ (ko) lungs than miR-200^+/+^(wt).

In order to evaluate the effect on downstream targets and associated pathways owing to loss of miR-200b on the lung tissue transcriptome, we performed Next Generation Sequencing (NGS) on total RNA samples from lungs of three 8-week-old miR-200b^−/−^ mice and three miR-200^+/+^ (wt) mice. Heat Map and unsupervised hierarchical clustering by sample and transcripts was performed on all samples passing QC using the top 500 genes that have the largest coefficient of variation based on FPKM counts (Fig. 4a). Table 1 shows the ten most differentially expressed mRNAs. The full list of differentially expressed transcripts is shown in Supplementary Table 3. We used Gene ontology (GO - Gene Ontology Consortium, 2000) enrichment analysis to identify GO terms that are significantly associated with differentially expressed protein coding genes. Using PANTHER Gene Ontology classification system^31^, we identified Notch and Wnt signalling among the most affected biological pathways and cytoskeletal as well as immunity proteins among the most affected protein class in miR-200b^−/−^ lungs compare to miR-200b^+/+^(Fig. 4b,c). We used r package topGO^32^ in order to generate Go network for Biogolical process and evaluate Biological function. Our current findings revealed that the three most affected biological processes in miR-200b^−/−^ mice lungs were related to cell cycle, apoptosis and protein transport (Fig. 4d). The most affected Biological Function is shown in Table 2. Among differentially expressed mRNAs, we confirmed the expression of *Plunc, Cyp2a5 and Cdh26* (palate lung and nasal epithelial clone) by q-PCR (Fig. 4e).

**Table 1:** The top 10 most significantly differentially expressed mRNAs, with log fold change (FPKM Log2_FC) between groups treatmentA (miR-200b^+/+^) and treatmentB (miR-200b^−/−^) with Benjamini-Hochberg FDR corrected q-values.

Gene_id	Gene	Locus	treatmentA	treatmentB	Log2_fold_change	Q_value
XLOC_008125	Lrrtm3	10:63430097–65003667	0.231458	0.00345736	−6.06493	0.0355741
XLOC_020864	Tcrg-C2	13:19304679–19311304	50.7836	1.27343	−5.31757	0.00634618
XLOC_044124	Bpifa1 or plunc	2:154142879–154149219	1353.78	76.2106	−4.15086	0.00634618
XLOC_069330	Slc5a11	7:123214779–123273253	0.0723419	1.11466	3.94563	0.00634618
XLOC_008308	Trpm2	10:77907721–77970563	17.5321	1.28117	−3.77447	0.00634618
XLOC_014662	Krt15	11:100131757–100135928	6.93844	0.507409	−3.77339	0.00634618
XLOC_022181	Thbs4	13:92751589–92794818	0.440093	0.033428	−3.71868	0.0340586
XLOC_014413	Spata20	11:94478903–94486179	0.0590388	0.773681	3.712	0.00634618
XLOC_067530	Cyp2a5	7:26835304–26952462	282.331	23.1095	−3.61083	0.00634618
XLOC_044638	Cdh26	2:178430530–178487366	4.61108	0.383838	−3.58654	0.00634618

**Table 2:** The significant GO terms for the genes found to be differentially expressed between treatmentA (wt) and treatmentB (ko) their corresponding annotation for Biological Function (BF).

GO.ID	Term	Annotated	Significant	Expected
GO:0015031	protein transport	1142	7	11.13
GO:0051301	cell division	435	6	4.24
GO:0006886	intracellular protein transport	653	5	6.36
GO:0046777	protein autophosphorylation	170	0	1.66
GO:0006915	apoptotic process	1336	29	13.02
GO:0007067	mitosis	306	3	2.98
GO:0006397	mRNA processing	322	2	3.14
GO:0008285	negative regulation of cell proliferation	451	5	4.39
GO:0001701	in utero embryonic development	374	3	3.64
GO:0043065	positive regulation of apoptotic process	295	10	2.87
GO:0000122	negative regulation of transcription from RNA polymerase II promoter	534	5	5.2
GO:0006281	DNA repair	357	0	3.48
GO:0043066	negative regulation of apoptotic process	499	16	4.86
GO:0045944	positive regulation of transcription from RNA polymerase II promoter	715	12	6.97
GO:0009968	negative regulation of signal transduction	692	10	6.74
GO:0045893	positive regulation of transcription, DNA-dependent	991	15	9.66
GO:0007049	cell cycle	1135	10	11.06
GO:0001666	response to hypoxia	225	11	2.19
GO:0006355	regulation of transcription, DNA-dependent	2844	31	27.71
GO:0006468	protein phosphorylation	1156	17	11.26

Annotated: Number of genes associated to the GO term, Significant: Number of significantly differentially expressed (p <= 0.05) genes within the annotated genes, Expected: Number of genes within the annotated genes that are expected to be significantly deferentially expressed (p < = 0.05) by random.

## Conclusions

We demonstrate for the first time that miR-200b plays a role in peripheral lung development by maintaining an epithelial cell phenotype. Our miR-200b deficient mice have lung function abnormalities, surfactant biophysical dysfunction with compromised pro-Surfactant Protein-C and surfactant protein-B expression. MiR-200b^−/−^ lungs have decreased distal airway branching, a denser lung parenchyma with thicker alveolar walls, a higher number of fibroblast-like cells and over-expression of a marker for EMT: Twist.

We generated miR-200b^−/−^ mice by targeted deletion of miR-200b in ES cells. We evaluated mature forms of all miR-200 family members to determine if there are any compensatory effects between the family members. Although miR-200a and miR-429 were expressed lower in miR-200b^−/−^ lungs, their expression was not undetectable like miR-200b. Based on our studies, we cannot exclude that downregulation of miR-200a and miR-429 contributed to the observed lung phenotype in miR-200b deficient mice. Moreover, miR-200c and miR-141, which are transcribed independently, are expressed normally suggesting that there are no compensatory effects.

Our miR-200b^−/−^ mice did not show any obvious breathing problems after birth, but lung function studies demonstrated severe peripheral airway obstruction in 8-week-old miR-200b^−/−^ mice. These observed lung function abnormalities can be due to lung surfactant deficiency or lung fibrosis. Using human fetal lung cultures, Benlhabib, *et al*. showed previously that miR-200 family members regulate epithelial type II cell differentiation and function^33^. They found that miR-200 family inhibitors down-regulated pro-SP-C and SP-B expression. Here, we show that miR-200b^−/−^ lungs have dysfunctional surfactant and compromised expression of these two proteins.

Using *in vivo* micro-CT scanning on alive animals, we found higher parenchymal density with significantly less air-filled distal alveoli in miR-200b^−/−^ mice. Our lung morphometry studies corroborated these results by showing that the percentage of airspace in miR-200b^−/−^ lungs was lower than in miR-200b^+/+^ lungs. Thus, both studies suggest that the observed peripheral airway obstruction is due to thicker alveolar walls reducing the airspace inside miR-200b^−/−^ lungs. Our immunofluorescence studies indicate that Vimentin and Twist expression are upregulated in the peripheral lung tissues of miR-200b^−/−^ mice. Interestingly, this was not confirmed in our NGS studies of miR-200b^−/−^ lungs. It has been shown that regulation of vimentin and twist expression are mainly at the translational level^34, 35^. Our data suggests that miR-200b is involved in the translation regulation of these genes in the lung. Taken together, the observed lung function abnormalities in miR-200b^−/−^ mice can be due to a mesenchymal-skewed, “fibrosis”-like lung phenotype. Others have suggested a role for miR-200b in lung fibrosis before^21^.

Using NGS analysis, we identified changes in the transcriptome in miR-200b^−/−^ lungs. Of note, we did not observe upregulation of the primary direct targets of the miR-200 family in the transcriptome analysis of miR-200b^−/−^ lungs. We hypothesize that this can be explained by the fact that most microRNAs directly regulate gene expression at the translational level. Therefore, changes in proteins of the direct gene targets of the miR-200 family might not be reflected in the transcriptome of miR-200b^−/−^ lungs. Gene ontology analysis showed that different signaling pathways are affected by miR-200b absence.

*Cyp2a5* (cytochrome P450, family 2, subfamily a, polypeptide 5) mRNA is one of the most down-regulated mRNAs in miR-200b^−/−^ lungs. Cytochrome P450s comprise a superfamily of enzymes crucial for metabolism of a diverse group of compounds, drugs and environmental pollutants. These enzymes are mainly present in the liver, but lower levels have been detected in the lung^36^. Down-regulation of CYP2A5 suggests the involvement of miR-200b in lung metabolism.

Palate lung and nasal epithelial clone (Plunc) is one of the mRNAs that was down-regulated more than four times in miR-200b^−/−^ lungs. PLUNC is the most abundant secretory protein in the lung and is expressed in nasal, oropharyngeal, and lung epithelial cells. Plunc acts as airway surfactant and plays a role in pulmonary host defence^37, 38^. It is essential for maintaining normal airway surface liquid homeostasis^39, 40^. Lower levels of *Plunc* can explain the increased elastance and airway resistance observed in the miR-200b^−/−^ lungs.

Cadherin-26 (CDH26) is another significantly down-regulated mRNA in the miR-200b^−/−^ lungs. Recently, others showed that CDH26 is involved in regulating lung epithelial cell polarity and differentiation. Knockdown of CDH26 results in a lack of epithelial cell polarity and differentiation^41^. Down-regulation of CDH26 in our knockout lungs can explain the fibroblast-like phenotype of the lungs in our knockout mice and the role of miR-200b in maintaining an epithelial cell phenotype.

Interestingly, these lungs still express the epithelial marker cytokeratin normally. These findings suggest a “partial EMT” in the lung parenchyma of miR-200b deficient mice. Others have shown that patients with idiopathic pulmonary fibrosis (IPF) have epithelial basal cells with a partial mesenchymal phenotype surrounding the fibroblastic foci^42^. Members of the miR-200 family are down-regulated in the lungs of patients with IPF and a mouse model of lung fibrosis^21^. We have previously discovered that lung hypoplasia in CDH lungs is characterized by reduced airway branching and a thickened interstitial mesenchymal cell layer recapitulating a fibrotic lung phenotype^4, 43^.

Knocking down miR-200b in our mice resulted in down-regulation of mature miR-200a and miR-429. These two miR-200 family members share the same transcript with miR-200b. This down-regulation can result from the removal of the miR-200b gene on the promotor activity or can be a post transcriptional effect of miR-200b on processing of the other microRNAs. Although miR-200c has the exact same seed sequence as miR-200b, the abundance of this microRNA along with miR141 was not changed in lungs and kidneys of 8-week old miR-200b^−/−^ mice suggesting the absence of any compensatory effects of these two miR-200 family members in miR-200b deficient mice. However, it is unclear at this point what the influence of downregulation of miR-200a and miR-429 on the lung phenotype of miR-200b deficient mice is.

Our *in vivo* studies demonstrate a new role for miR-200b in distal lung airway development by regulating epithelial and fibroblast cell differentiation.

## Materials and Methods

### Ethics and animal work

Mice were maintained in accordance with the guidelines of “*Guide to the Care and Use of Experimental Animals”*. The *in vivo* experiments were approved by the Animal Research Review Committee at the University of Manitoba.

### Generation of C57BL/6; miR-200b^tm1.1(NCOM)MFGC^ mice

We generated a miR-200b^−/−^ (KO) mouse by replacing the complete miR-200b gene by targeted homologous recombination in C57Bl/6N mouse embryonic stem cells using the NorCOMM knockout cassette (online data supplement).

### LacZ staining

Whole mouse embryos are stained for β-galactosidase (lacZ) activity using X-gal as described before^44^. For E14.5 and older, we used a razor blade to section the embryos in half to facilitate penetration.

### Lung Mechanics

Eight-week-old male miR-200b^+/+^(wt) or miR-200b^−/−^ (ko) mice (at least six mice for each group) were anesthetized with intra-peritoneal sodium pentobarbital (90 mg/kg). The trachea was dissected using fine dissection scissors and a 20-gauge polyethylene catheter was inserted which was further connected to a flexiVent small animal ventilator (Scireq Inc. Montreal). Mice were ventilated with a tidal volume of 10 ml/kg body weight, 150 times per minute. A positive end expiratory pressure (PEEP) of 3 cmH_2_O was used for all studies. Mice were subjected to an increased dose of nebulized methacholine (MCh) challenge protocol to assess concentration response characteristics of respiratory mechanics. For MCh challenge, ~30 μL of saline containing from 0 to 50 mg/ml MCh was delivered over 10 seconds using an in-line ultrasonic nebulizer. To assess the effects of MCh challenge on respiratory mechanics we used low frequency forced oscillations (1–20 Hz). Respiratory mechanical input impedance (*Zrs*) was derived from the displacement of the ventilator’s piston and the pressure in its cylinder. Correction for gas compressibility, and resistive and accelerative losses in ventilator, tubing and catheter were performed per the manufacturer instructions, using dynamic calibration data obtained from snap shot perturbation applied to the system in an open and closed configuration. By fitting *Zrs* to the constant phase model and Prime-3 perturbation flexiVent software calculated conducting airway resistance known as Newtonian resistance (R_n_), peripheral tissue/airway resistance known as tissue damping (G) and tissue elastance or stiffness (H). Values for each parameter were calculated as the mean of all 12 perturbation cycles performed after each MCh challenge.

### Surfactant Biophysical Properties

Bronchoalveolar lavage fluid (BALF) was collected with 4 repeated washes of excised lungs using 2 ml saline in total. The fresh supernatant was used for assessing biophysical surfactant function using a capillary surfactometer according to the manufacture’s protocol (Calmia Medical, Inc.). We used 3 miR-200b^−/−^ male mice and 3 miR-200b^+/+^ for this measurement and each sample was measured 5 times.

### Micro-CT scans

Three miR-200b^−/−^ and three miR-200b^+/+^ male mice were anesthetized in an anesthetic chamber with 5% isoflurane and scanned for 32 minutes, while the mice were breathing normally under an anesthetic mask, using the SkyScan 1176 x-ray microtomography system equipped with a large format 11 megapixel x-ray camera (Small animal model imaging core facility, University of Manitoba). Images were acquired at 18 μm resolution with an exposure time of 310 ms and 0.5° rotation step using a 0.5 mm aluminum filter and source current and voltage of 500 μA and 50 kV respectively. Images were reconstructed using NRecon (Bruker MicroCT, Kontich, Belgium) with the dynamic range set to the same values for each mouse (0–0.08) and a beam hardening correction of 30%. Reconstructed images were processed using CTan (Bruker MicroCT, Kontich, Belgium) to visualize airways. To ensure that the same region of tissue was used for comparison between mice, the slice showing initial branching of the trachea into bronchi was found and the first slice for analysis was set to be 3.25 mm (181 slices) above this slice. The last slice included in the analysis was set to the slice corresponding to the base of the lungs (i.e. the first slice showing only diaphragm and no lung tissue). Image size was reduced by drawing a region of interest around the lung tissue, excluding muscle and bone, and saving the reduced volume within the region of interest between the upper and lower slice limits. Slices were then loaded in CTVox (Bruker MicroCT, Kontich, Belgium) as minimum intensity projection images. The reduced data set was further processed in CTan to allow calculation of airspace volume and structure thickness. The 8-bit images were binarised using a lower threshold of 15 and an upper threshold of 255 to segment tissue containing air from surrounding tissues. An ROI shrink-wrap was performed in 2D followed by the bitwise operation image = (image) XOR (ROI) to produce an image of the air-containing tissue. This image was then de-speckled to remove white speckles smaller than 15 voxels. The volume of the segmented regions was calculated to produce an estimate of the volume of air in the lungs.

### Measuring area percentage of airspace

Three miR-200b^−/−^ and three miR-200b^+/+^ 8-week-old lungs were inflation-fixated and embedded in paraffin. After performing H&E staining on three sections per lungs, we scanned the whole sections using an Axio Scan.Z1 microscope from Zeiss. We measured area percentage of the airspace using ZEN Image Analysis software and a module based on color coding the tissue and empty areas (airspace). Using the software, we eliminated the large airways from the calculation (Supplementary Fig. 7).

### Tissue Immunofluorescent Staining

Three 8-week-old miR-200b^−/−^ or miR-200b^+/+^ lungs were inflated intratracheally with 1 ml of 10% formalin via the cannula by gentle infusion. All tissues were fixed in 10% formalin for overnight. Lungs were embedded in paraffin after dehydration in a graded ethanol series followed by xylene. Immunofluorescence was performed on 5 μm sections using vimentin (Abcam; ab92547; 1:800), pro-Surfactant Protein-C (Abcam; ab40879; 1:800), pro + mature Surfactant Protein-B (Abcam; ab40876, 1:50) and twist (Abcam; ab50887; 1:200) antibodies as previously described^45^.

### Cell Immunofluorescent Staining

Human bronchial epithelial cells, BEAS-2B (ATCC, Manassas, VA, USA), cells were transfected with 0.01 μg/ml of LNA-hsa-miR200b inhibitor or negative control oligonucleotides Exiqon, Denmark). After 48 h cell were fixed with 4% PFA and immunofluorescence was performed using vimentin (Abcam; ab92547; 1:800) and cytokeratin (BioLegend, 628602, 1:200).

### Fetal lung explant culture

For mice lung explant culture, lungs were isolated from E11.5 embryos (offspring from a miR-200b ^+/−^ cross) and transferred to porous membranes (IsoporeTM) filters with dimensions of 1 mm × 1.5 mm pore size (Millipore, USA) in a 12-well plate for a semidry floating explant culture and cultured for four days in a 1:1 mixture of DMEM and Ham’s F-12 Nutrient supplemented with 100 μg/ml streptomycin, 100 units/ml penicillin, 0.25 mg/ml ascorbic acid. Branching morphogenesis and epithelial perimeter length were monitored daily in all groups by stereomicroscopy, photographs taken and measurements performed using ImageJ software. The difference between day 0 (D0: 0 hours) and day 4 (D4: 96 hours) of culture, were expressed as D4/D0 ratio.

### Scratch (wound healing) assay

The scratch (wound healing) or migration assay was performed as previously described by others^46^. Briefly, BEAS-2B cells were plated in 12 well plates and transfected with 0.01 μg/ml of LNA-hsa-miR200b inhibitors or negative control oligonucleotides. After 12 hours, we scraped the cell monolayer in a straight line with a 200-µl pipette tip. The cell cultures were washed with PBS to remove the debris followed by transfection with miR-200b inhibitors. Cell migration was monitored and photographed over time. Distance travelled from the initial scratch site was measured after 18 h and the migration distance was quantified with ImageJ software.

### Library preparation and Next Generation Sequencing

The library preparation was done using the TruSeq® Stranded mRNA Sample preparation kit (Illumina inc). The starting material of total RNA (100 ng) was mRNA enriched using the oligodT bead system. The isolated mRNA was subsequently fragmented using enzymatic fragmentation. Then first strand synthesis and second strand synthesis were performed and the double stranded cDNA was purified (AMPure XP, Beckman Coulter). The cDNA was end repaired, 3′ adenylated and Illumina sequencing adaptors were ligated onto the fragment ends, and the library was purified (AMPure XP). The mRNA stranded libraries were pre-amplified with PCR and purified (AMPure XP). The libraries size distribution was validated and quality inspected on a Bioanalyzer high sensitivity DNA chip (Agilent Technologies). High quality libraries were quantified using qPCR, the concentration normalized and the samples pooled according to the project specification (number of reads). The library pool(s) were re-quantified with qPCR and optimal concentration of the library pool used to generate the clusters on the surface of a flowcell before sequencing using Nextseq500/ High Output sequencing kit (51 cycles according to the manufacturer instructions (Illumina Inc.). All experiments were conducted at Exiqon Services, Denmark.

### Statistical analysis

All data are presented as mean +/− standard error of mean, from a minimum of three independent experiments. Statistical significance was determined by two-way ANOVA or by *student’s t*-test as indicated in the figure legends. A p-value ≤ 0.05 was considered significant.

## Electronic supplementary material

Supplementary Information
