# Supplementary material for: MicroRNA-200b regulates distal airway development by maintaining epithelial integrity
Source: Sci Rep. 2017 Jul 25;7:6382. doi: 10.1038/s41598-017-05412-y (PMC5526907; doi:10.1038/s41598-017-05412-y)
Supplement: Supplementary file 1 — Supplementary Information [file 41598_2017_5412_MOESM1_ESM.docx]

**Online Data Supplement**

**MicroRNA-200b regulates distal airway development by maintaining epithelial integrity**

Naghmeh Khoshgoo^1,2,3^, Robin Visser^1,2^, Landon Falk^1,2^, Chelsea Arlana Day^1,2^, Dustin Ameis^1,2^, Barbara M. Iwasiow^1,2^, Fuqin Zhu^1,2^, Arzu Öztürk^4,5^, Sujata Basu^1,3^, Molly Pind^4,5^, Agnes Fresnosa^4,5^, Mike Jackson^6^, Vinaya Kumar Siragam^1,2^, Gerald Stelmack^1,3^, Geoffrey G. Hicks^4,5^, Andrew Halayko^1,3^, Richard Keijzer^1,2,3 *^

**Supplementary Methods:**

**Generation of C57BL/6; miR-200b^tm1.1(NCOM)MFGC^ mice**

**Vector Design and Construction:** The mouse miR-200 locus is comprised of three microRNA genes, mmu-mir-200b, mmu-mir-200a and mmu-mir-429, in the first intron of the *Ttll10* gene of mouse chromosome 4. The targeting vector was designed strategically to target the deletion of only miR-200b (ENSMUSG00000065549) within the cluster and to avoid any changes to miR-200a, miR-429 or any other conservative regions within the locus. The miR-200b targeting vector was constructed utilizing the pGOHANU vector, which contains the F3/FRT flanked NORCOMM targeting cassette comprised of three functional units: the SA-IRES-LacZ-pA genetrap reporter, a loxP-flanked hβactP-∆TK1-T2A-Neo-pA selection cassette, and an AttP-Puro-pA docking cassette ^1,2^ (Fig. 1a). The docking cassette was designed to facilitate docking applications with ØC31 integrase in ES cells to modify a given targeted allele ^3^ and was not used in this study. Assembly of the final targeting vector included an 11 kb mus musculus genomic region containing miR-200b on chromosome 4 (155429790 – 155429859/ negative strand) using the NORCOMM recombinering strategy modified from that developed by EUCOMM ^4^. Briefly, the identified C57BL/6 BAC clones, RP23-118E21, RP23-382A8, RP23-350D2, RP23-139J18, were used to assemble intermediate and final targeting vectors according to the Sanger Design ID #373799. The region was designed such that the 2891 bp 5’ homology arm (155429953 – 155432844) and the 6595 bp 3’ homology arm (155423096 – 155429691) define the points of homologous recombination (Fig. 1b). In the process, intervening sequences contain miR-200b genomic sequences were replaced by the NORCOMM cassette (F3-SA-IRES-LacZ-pA-loxP-hβactP-∆TK1-T2A-Neo-pA-loxP-AttP-Puro-pA-FRT). The final targeting vector was created by use of a three-way gateway (Invitrogen) mediated *in vitro* recombination sub-cloning strategy. Final targeting vectors were sequence validated across junction points of subcloned arms, and across the site-specific recombination elements, loxP, F3, FRT and AttP-docking sites.

**ES cell targeting and PCR genotyping of targeted miR-200b allele:** The miR-200b knock out mice were generated by replacement of the endogenous miR-200b with the NorCOMM cassette using a homologous recombination targeting strategy (Fig. 1b) ^5^. Briefly, 10^7^ C2 embryonic stem (ES) cells (C57BL/6NTac), were electroporated using 2.5 µg of linearized targeting vector with AsiSI restriction enzyme. Electroporated ES cells were cultured ten days in G418 (neo) selection (100 μg/ml) over neomycin resistant mouse embryo fibroblast feeder cells derived from TgN(DR4)1Jae/J mice. 32 healthy neomycin resistant ES colonies were picked and cloned cells were expanded in 96 well plate format for either genomic DNA isolation or cryopreservation. Correctly targeted ES cells were identified by long range PCR screening of the 5’ and 3’ homology arms using primers spanning from the NorCOMM cassette to a site beyond the end of the respective homology arms. ES clones that had undergone correct recombination would have a 5’ homology arm PCR product of 3356 bp (primers GH2871 and GH717, Fig. 1b G5). Four 5’ homology arm sequence validated clones were next screened to identify the integrity of the 3’ homology arm by producing a correct PCR product size of 6979 bp (primers GH1300 and GH2892, Fig. 1b G3). Both amplified PCR products were subjected to sequencing across the ends of the homology arms to validate correct and precise recombination events. PCR-sequencing was also used to validate the integrity of the F3, FRT and loxP sites in the NorCOMM cassette to ensure downstream applications of *in vivo* site-specific recombination (Fig. 1b). Three of four ES clones (A9, B9, F9) were fully sequence validated and validated by Southern blotting (data not shown).

**Generation of miR-200b knock out transgenic mice:** MiR-200b knock out F9 ES clone was used to generate chimeras (F0) by ES cell injection into B6(Cg)Tyr ^c-2j^/J blastocysts, as previously described ^6^, resulting in chimeras with greater than 80% black coat color chimerism. Male chimeras were mated with C57BL/6N females to derive mice with germ line transmission (GLT) of the miR-200b KO allele. This line was designated as miR-200b^tm1(NCOM)MFGC^. Removal of the hβact promoter driven ∆TK1-T2A-neomycin cassette was performed *in vivo* by mating male miR-200b^tm1(NCOM)MFGC^ mice harboring the miR-200b KO allele with female B6.C-TG(CMV-Cre)1Cgn/J transgenic mice, in which Cre recombinase is constitutively expressed (The Jackson Laboratory, #006054). The resultant miR-200b Cre-excised allele, miR200b^tm1.1(NCOM)MFGC^ (Fig. 1c), was sequence validated following PCR analysis across the remaining loxP site (primers GH3617 and GH3618). Loss of the neomycin selection cassette was also verified by PCR (primers GH3619 and GH3620). Elimination of the transgenic Cre-recombinase allele was selected for during routine breeding and genotyping. The resulting miR-200b^tm1.1(NCOM)MFGC^ mice on a homogenous C57BL/6N background were used for all experiments in the present study. Mice were genotyped from ear biopsies using a multiplex PCR strategy (primers GH3369 and GH3370 for 494 bp wild type allele product, and primers GH3369 and GH717 for 390 bp knock out allele product) under the following conditions: 98°C 2 min, 98°C 10 sec, 64°C 30 sec, 72°C 45 sec, Cycle to Step 2 another 32 times, 72°C for 5 min, 8°C to Hold (Supplementary Table 1).

**RNA extraction and qPCR**

lungs were isolated in icecold PBS, snap frozen in liquid nitrogen and stored at -80°C until processed. Total RNA was extracted using the miRCURY™ Isolation Kit (Ambion), according to the manufacturer's instructions. For microRNA analysis, cDNA was randomly primed from 20 ng total RNA using the Exiqon cDNA synthesis kit. RT-qPCR was subsequently performed using locked nucleotide acid (LNA) primers for miR-200 family members and miR-103 (as an endogeneous control). All primer sequences are provided in the Supplementary Information (Supplementary Table 2). We used the miRCURY LNA™ Universal RT microRNA PCR protocol (Exiqon) in a total reaction volume of 20µl. Briefly, RT-qPCR was performed in triplicate with a 1:80 dilution of cDNA using the SYBR green PCR system on an ABI 7500 Real-Time PCR machine (Applied Biosciences). Data were collected and analyzed using ABI 7500 v1.4.0 software (Applied Biosciences). MicroRNA expression levels were determined using the relative quantification feature of the ABI 7500 v1.4.0 software.

**Fetal lung explant culture**

For mice lung explant culture, lungs were isolated from E11.5 embryos (offspring from a miR-200b +/- cross) and transferred to porous membranes (IsoporeTM) filters with dimensions of 1 mm x 1.5 mm pore size (Millipore, USA) in a 12-well plate for a semidry floating explant culture and cultured for four days in a 1:1 mixture of DMEM and Ham's F-12 Nutrient supplemented with 100 μg/ml streptomycin, 100 units/ml penicillin, 0.25 mg/ml ascorbic acid. Branching morphogenesis and epithelial perimeter length were monitored daily in all groups by stereomicroscopy, photographs taken and measurements performed using ImageJ software. The difference between day 0 (D0: 0 hours) and day 4 (D4: 96 hours) of culture, were expressed as D4/D0 ratio.

**Supplementary results**

**Supplementary Fig. 1.** PCR & Sequence Validation. Following gene targeting, neomycin-resistant ES cell clones were selected with G418 and subjected to rigorous PCR and sequenced-based analysis to confirm the correct targeting of miR-200b. Correct sized long range PCR products spanning from gene-specific primers outside the homologous arms of the targeting vector to vector-specific primers within the NorCOMM cassette confirmed correct targeting of miR-200b. Sequence analysis of long range PCR products across the genomic junction spanning the ES cell genomic loci and the genomic region of the homology arms within the original targeting vector definitively confirm miR-200b targeting (PCR products – blue lines in panel A; sequencing primers G5 and G3 site relative primers are indicated by blue arrows). Similarly, sequence analysis of PCR product spanning the junction between the genomic homology arms and the integrity of the functional elements of the targeting vector (F3 and FRT sequencing primers highlighted) were confirmed with Southern Blot. Summary of data shows three miR-200b knockout ES clones are fully validated and utilized for generation of miR200b^tm1(NCOM)MFGC^ of germ line transmitted mice.

**Supplementary Fig. 2.** Absence of miR-200b expression in miR-200b knockout lung explants was confirmed using RT-qPCR. **P* <0.05, ***P* <0.01, ****P* <0.001, one-way ANOVA. Data represent the mean of three independent experiments.

**
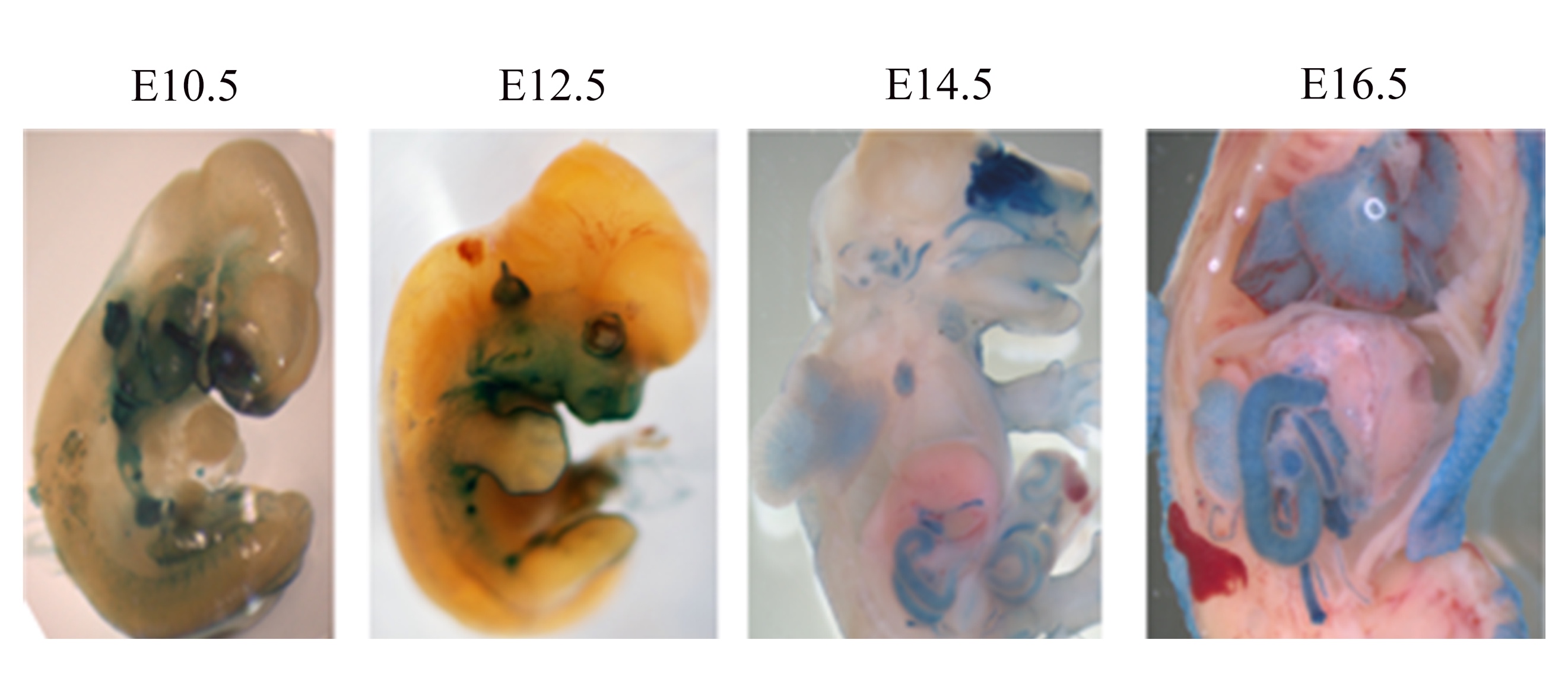
**

Gastrointestinal tract

& lung bud

Otic Vesicle

Mammary Bud

Palate

**Supplementary Fig. 3.** Whole mount lacZ expression in miR-200b ko embryos at E10.5, E12.5 and E14.5. LacZ staining (blue) is observed in the lungs, palate, otic vesicle and mammary buds.

 **Supplementary Fig. 4.** MiR-200b ko mice have significantly lower Hysteresivity before methacholine challenge, at 3, 12 and 50 mg/ml of MCh. Also, miR-200b +/- mice have lower hysteresivity at 6, 12 and 25 mg/ml of MCh as well. Comparisons were made to wildtype. ^#^*P* <0.05, ^##^*P* <0.01, ^###^*P* <0.001, **P* <0.05, ***P* <0.01, ****P* <0.001, two-way ANOVA. Data represent the mean of at least 6 independent experiments.

**Supplementary Fig. 5.** RT-qPCR for all miR-200 family members on lungs from 8-week-old mice using LNA primers. miR-200b absence was confirmed. miR-200a and miR-429 were significantly downregulated but no changes were observed in abundance of miR-200c and miR-141 ***P* <0.01, Student’s t-test, Data represent mean ± SEM of at least four independent experiments.

**Supplementary Fig. 6.** Absence of miR-200b expression and lower expression of miR-200a and miR-429 in kidneys of miR-200b knockout mice was confirmed using RT-qPCR.

**a b**

**
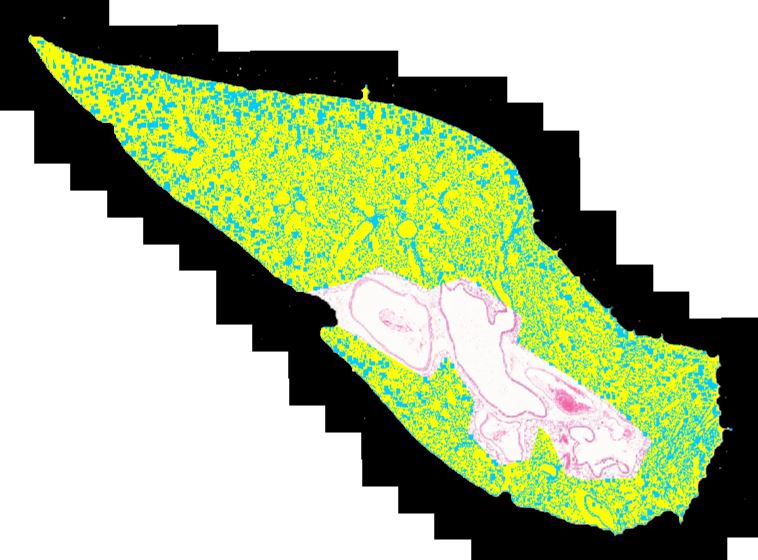

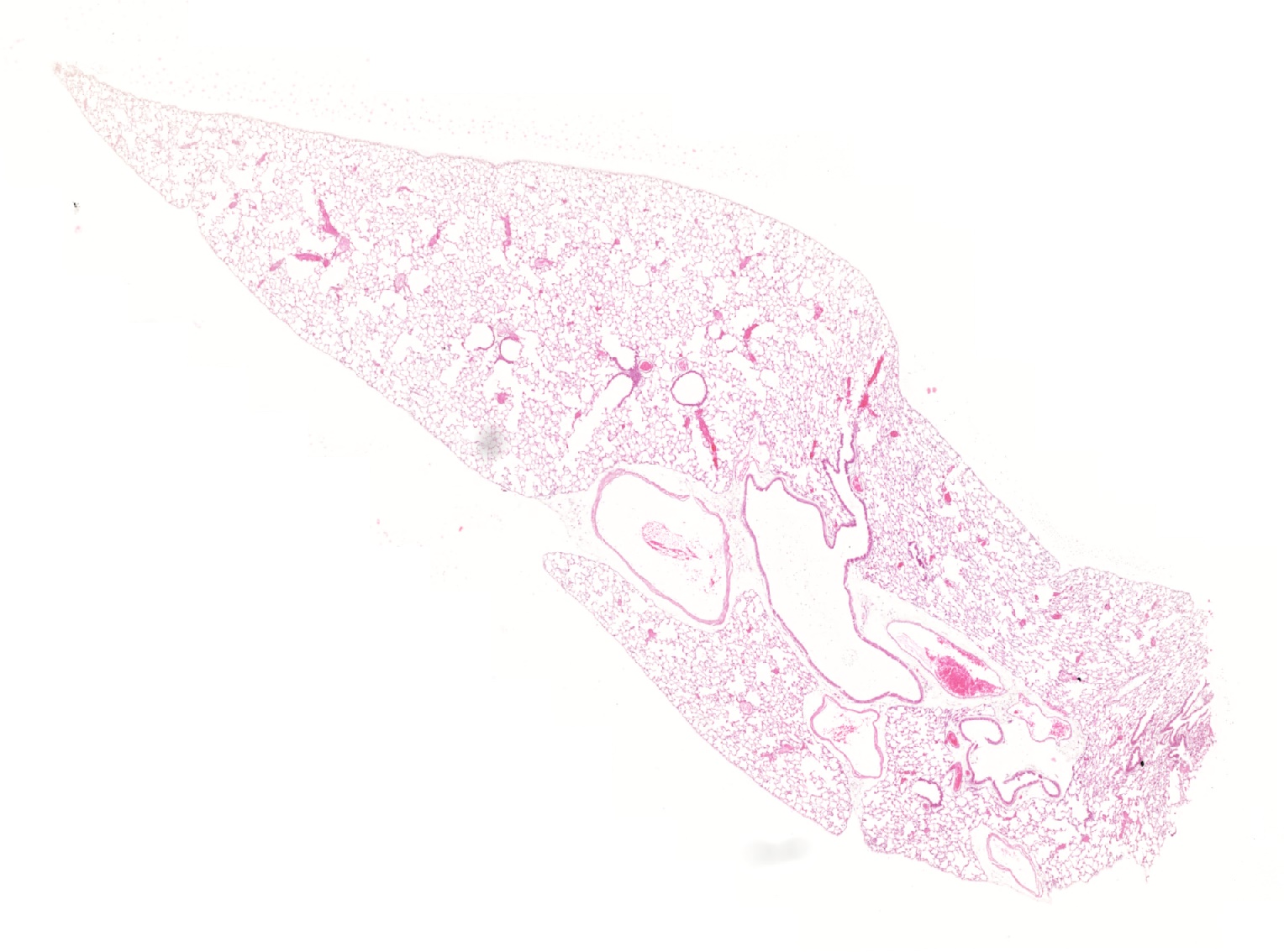
**

**Supplementary Fig. 7. Are percentage of airspace measurement.** Example of tissue scanned by Zeiss Laser Scanning Microscope (a) and We measured area percentage of the airspace using ZEN Image Analysis software and module (base on color coding the tissue and empty areas (airspace)). Using the software, we could eliminate the large airways from the calculation (b)

**Supplementary Tables:**

| **Primer** | **Assay** | **Primer type** | **5’ to 3’ sequence** | **Product size** |
| --- | --- | --- | --- | --- |
| GH2871 | 5' homology arm validation | Forward | CTTAAAGCAGCCACTGCTGTTCC | 3356 bp |
| GH717 | 5' homology arm validation | Reverse | CACCGACGCCAATCACAAACAC |  |
| G5 (GH2883) | 5' homology arm sequencing | Sequencing | GGGCATGAAGATCTCGTCTCTGT | – |
| GH1300 | 3' homology arm validation | Forward | TCTTATCATGTCTGCTCGAAGC | 6979bp |
| GH2892 | 3' homology arm validation | Reverse | TGAAGGTCAAAGAAGCTCCAAGC |  |
| G3 (GH2976) | 3' homology arm sequencing | Sequencing | ATTTGGCCTTTCTTTGCTGTCAG | – |
| GH1343 | F3 validation | Forward | CGCATAACGATACCACGATATCAAC | 1402bp |
| GH1177 | F3 validation | Reverse | ACAGTATCGGCCTCAGGAAGATC |  |
| F3 (GH681) | F3 Sequencing | Sequencing | GTAAGTCGATATGTTTATTCTTC | – |
| GH1353 | FRT validation | Forward | TGAATGGAAGGATTGGAGCTACG | 1490bp |
| GH1345 | FRT validation | Reverse | TACTGCGACTATAGAGATATCAACC |  |
| FRT (GH809) | FRT sequencing | Sequencing | CTGCATTCTAGTTGTGGTTTGTCC | – |
| GH3617 | ∆TK-T2A-Neo cassette excision | Forward | CCCGTCAGTATCGGCGGAAT | 659 bp |
| GH3618 | ∆TK-T2A-Neo cassette excision | Reverse | ACCCACACCTTGCCGATGTC |  |
| GH3619 | check neo specific loss | Forward | GGAAGGGACTGGCTGCTATTGG | 521 bp |
| GH3620 | check neo specific loss | Reverse | TCAAGAAGGCGATAGAAGGCGATG |  |
| GH3370 | Genotyping WT allele | Reverse | CCCATAGCCCTACCTTGGATAAGG | 494 bp |
| GH717 | Genotyping Mut allele | Reverse | CACCGACGCCAATCACAAACAC | 390 bp |
| GH3369 | Genotyping common | Forward | AGGGGAACTTGTCTATGGCCATG |  |

**Supplementary Table 1:** Primers used for genotyping or Sequencing of miR-200b ^+/+, -/+ or -/-^

| LNA microRNA primer set | Description |
| --- | --- |
| U6 snRNA (hsa, rno,mmu) | endogenous control |
| miR-103 | endogenous control |
| hsa-miR-200b | tested microRNA |
| hsa-miR-200a | tested microRNA |
| hsa-miR-429 | tested microRNA |

**Supplementary Table 2:** Locked nucleic acid (LNA) primer sets used in real-time quantitative PCR (RT-qPCR)

| Gene_id | Gene | Locus | Treatment A | Treatment B | | Log2_fold_change | | q_value |
| --- | --- | --- | --- | --- | --- | --- | --- | --- |
| XLOC_079375 | - | 9:18558358-18571556 | 3.54387 | | 279.112 | | 6.29938 | 0.00634618 |
| XLOC_008125 | Lrrtm3 | 10:63430097-65003667 | 0.231458 | | 0.00345736 | | -6.06493 | 0.0355741 |
| XLOC_046320 | Gm10800 | 2:98666546-98667301 | 31.0653 | | 0.467457 | | -6.05432 | 0.00634618 |
| XLOC_020864 | Tcrg-C2 | 13:19304679-19311304 | 50.7836 | | 1.27343 | | -5.31757 | 0.00634618 |
| XLOC_023499 | Trac | 14:54187894-54224201 | 1272.67 | | 35.2599 | | -5.17369 | 0.00634618 |
| XLOC_080741 | AC163666.1,SNORD50 | 9:88595233-88599516 | 0.913589 | | 29.5595 | | 5.01593 | 0.00634618 |
| XLOC_047434 | Gm14221 | 2:160568378-160619973 | 2.88636 | | 55.1965 | | 4.25725 | 0.00634618 |
| XLOC_044124 | Bpifa1 | 2:154142879-154149219 | 1353.78 | | 76.2106 | | -4.15086 | 0.00634618 |
| XLOC_069330 | Slc5a11 | 7:123214779-123273253 | 0.0723419 | | 1.11466 | | 3.94563 | 0.00634618 |
| XLOC_061800 | - | 5:137178738-137180434 | 1.87136 | | 0.124166 | | -3.91375 | 0.00634618 |
| XLOC_008308 | Trpm2 | 10:77907721-77970563 | 17.5321 | | 1.28117 | | -3.77447 | 0.00634618 |
| XLOC_014662 | Krt15 | 11:100131757-100135928 | 6.93844 | | 0.507409 | | -3.77339 | 0.00634618 |
| XLOC_022181 | Thbs4 | 13:92751589-92794818 | 0.440093 | | 0.033428 | | -3.71868 | 0.0340586 |
| XLOC_014413 | Spata20 | 11:94478903-94486179 | 0.0590388 | | 0.773681 | | 3.712 | 0.00634618 |
| XLOC_069418 | Doc2a | 7:126847415-126865377 | 0.542942 | | 0.0442615 | | -3.61667 | 0.00874121 |
| XLOC_067530 | Cyp2a5 | 7:26835304-26952462 | 282.331 | | 23.1095 | | -3.61083 | 0.00634618 |
| XLOC_044638 | Cdh26 | 2:178430530-178487366 | 4.61108 | | 0.383838 | | -3.58654 | 0.00634618 |
| XLOC_035369 | 1110038B12Rik,SNORD48,Snord52,snR78 | 17:34950237-34952471 | 151.769 | | 13.8386 | | -3.45511 | 0.00634618 |
| XLOC_027393 | - | 15:99875641-99879598 | 8.49763 | | 0.803171 | | -3.40328 | 0.00634618 |
| XLOC_077137 | Taf1d | 9:15283336-15316913 | 134.224 | | 14.274 | | -3.23319 | 0.00634618 |
| XLOC_006541 | Slc5a8 | 10:88885991-88929505 | 1.14442 | | 0.123307 | | -3.21428 | 0.00634618 |
| XLOC_063698 | Mitf | 6:97807057-98021349 | 3.20994 | | 26.4442 | | 3.04234 | 0.00634618 |
| XLOC_080590 | 7SK | 9:78175302-78175633 | 21.3643 | | 2.62084 | | -3.0271 | 0.00634618 |
| XLOC_011029 | Dvl2 | 11:70000594-70015411 | 54.2064 | | 6.88511 | | -2.97691 | 0.00634618 |
| XLOC_001898 | Myoc | 1:162639149-162658173 | 1.38254 | | 0.197349 | | -2.8085 | 0.00634618 |
| XLOC_065652 | Reg3g | 6:78466268-78468872 | 214.689 | | 31.3487 | | -2.77577 | 0.00634618 |
| XLOC_015248 | Sectm1b | 11:121053457-121063569 | 0.868546 | | 0.127885 | | -2.76375 | 0.0233719 |
| XLOC_057273 | Gm13054 | 4:148000721-148004014 | 6.76491 | | 1.06767 | | -2.6636 | 0.00686653 |
| XLOC_004449 | Fmo6 | 1:162916550-162937225 | 1.52488 | | 0.257978 | | -2.56337 | 0.00634618 |
| XLOC_053781 | Cyp4a12b | 4:115411623-115439034 | 2.13431 | | 0.381192 | | -2.48518 | 0.00634618 |
| XLOC_060723 | Cnga1 | 5:72603695-72642752 | 0.311571 | | 1.65699 | | 2.41093 | 0.00634618 |
| XLOC_049689 | Col11a1 | 3:114030539-114220718 | 0.562002 | | 0.109728 | | -2.35664 | 0.0187494 |
| XLOC_069920 | Sbk2 | 7:4957080-4964348 | 2.42296 | | 0.479149 | | -2.33822 | 0.00791765 |
| XLOC_077940 | Cyp1a1 | 9:57697602-57703823 | 51.9639 | | 10.7954 | | -2.2671 | 0.00634618 |
| XLOC_034299 | - | 17:80896864-80928497 | 12.6891 | | 61.0005 | | 2.26523 | 0.00634618 |
| XLOC_066565 | BC048546 | 6:128539821-128581606 | 9.52031 | | 2.0065 | | -2.24633 | 0.00634618 |
| XLOC_043654 | Nop56 | 2:130274429-130284547 | 62.102 | | 284.344 | | 2.19493 | 0.00634618 |
| XLOC_037500 | Cidea | 18:67321208-67367794 | 3.7549 | | 0.823973 | | -2.18811 | 0.00634618 |
| XLOC_035686 | Capn11 | 17:45630203-45659325 | 0.941383 | | 0.207637 | | -2.18071 | 0.00634618 |
| XLOC_016822 | Serpina3k,Serpina3m | 12:104338485-104394257 | 1.956 | | 0.45524 | | -2.10321 | 0.00634618 |
| XLOC_053986 | Rhbdl2 | 4:123787873-123830013 | 1.48329 | | 0.357287 | | -2.05365 | 0.00634618 |
| XLOC_010602 | Acsl6 | 11:54303797-54364756 | 0.402241 | | 0.0997272 | | -2.012 | 0.0408845 |
| XLOC_073489 | Ank1 | 8:22974843-23150497 | 6.75914 | | 1.68162 | | -2.00699 | 0.00634618 |
| XLOC_063058 | AC140385.1 | 6:67896176-67896642 | 4.02257 | | 15.6759 | | 1.96236 | 0.00634618 |
| XLOC_044533 | Pck1 | 2:173153047-173159273 | 9.55376 | | 2.45353 | | -1.96121 | 0.0210639 |
| XLOC_030754 | Mrap | 16:90738323-90749785 | 3.03823 | | 0.813923 | | -1.90027 | 0.00634618 |
| XLOC_060891 | Sult1d1 | 5:87554644-87569027 | 26.7668 | | 7.17198 | | -1.9 | 0.00634618 |
| XLOC_059565 | Azgp1 | 5:137981520-137990233 | 3.74742 | | 1.00488 | | -1.89887 | 0.00634618 |
| XLOC_057464 | Ttll10 | 4:156034608-156059802 | 5.55515 | | 1.51785 | | -1.8718 | 0.00634618 |
| XLOC_025205 | Mcpt4 | 14:56059743-56062310 | 1.996 | | 0.554963 | | -1.84665 | 0.0117784 |

**Supplementary Table 3:**  Table of full list of significantly differentially expressed mRNAs, with log fold change (FPKM Log2_FC) between groups treatment (wt) and treatment (miR-200b^-/-^) with Benjamini-Hochberg FDR corrected q-values.

| Parameters | Description |
| --- | --- |
| Source Voltage | 50 kV |
| Source Current | 500 μA |
| Image Pixel Size | 9 μm |
| Filter | 0.5 mm AI |
| Exposure | 1240 ms |
| Rotation Step | 0.500 deg |
| Frame Averaging | ON (3) |
| Scan duration | 00:32:25 |

**Supplementary Table 4:**  Important parameters used in SkyScan1176 for the in vivo lung micro-CT scans

**References:**

1. Austin, C. P. *et al.* The knockout mouse project. *Nat. Genet.* **36,** 921–4 (2004).

2. Bradley, A. *et al.* The mammalian gene function resource: The International Knockout Mouse Consortium. *Mamm. Genome* **23,** 580–586 (2012).

3. Monetti, C. *et al.* PhiC31 integrase facilitates genetic approaches combining multiple recombinases. *Methods* **53,** 380–385 (2011).

4. Skarnes, W. C. *et al.* A conditional knockout resource for the genome-wide study of mouse gene function. *Nature* **474,** 337–42 (2011).

5. Gertsenstein, M. *et al.* Efficient generation of germ line transmitting chimeras from C57BL/6N ES cells by aggregation with outbred host embryos. *PLoS One* **5,** 1–8 (2010).

6. Hicks, G. G. *et al.* Fus deficiency in mice results in defective B-lymphocyte development and activation, high levels of chromosomal instability and perinatal death. *Nat. Genet.* **24,** 175–179 (2000).
